# Investigation of Immediate Postoperative Pain following Orthognathic Surgery

**DOI:** 10.1155/2021/9942808

**Published:** 2021-05-31

**Authors:** Han-Jen Hsu, Kun-Jung Hsu

**Affiliations:** ^1^Department of Oral and Maxillofacial Surgery, Kaohsiung Medical University Hospital, Kaohsiung Medical University, Taiwan; ^2^School of Dentistry, College of Dental Medicine, Kaohsiung Medical University, Kaohsiung, Taiwan; ^3^Department of Dentistry, Kaohsiung Municipal Ta-Tung Hospital, Kaohsiung, Taiwan

## Abstract

**Purpose:**

The purpose of this study was to compare postintervention pain related to orthodontic treatment and orthognathic surgery. *Material and Methods*. One hundred patients who received only orthodontic treatment are the nonsurgical group. One hundred other patients were separated equally into the following four orthognathic surgical subgroups. The visual analog scale (VAS) score was used to measure postoperative pain. Patient- and operation-related factors were compared among the four surgical subgroups. The null hypothesis was that there was no difference between orthodontic treatment and orthognathic surgery in terms of posttreatment pain.

**Results:**

There were no significant differences between the nonsurgical and surgical groups for gender (*P* = 0.780) or age (*P* = 0.473). The VAS scores of the nonsurgical group (mean: 3.59) were significantly (*P* = 0.007) higher than those of the surgical group (mean: 3.06). The null hypothesis was rejected. Within the surgical subgroups, no significant differences were observed between the men and women for age, operation time, blood loss volume, or blood laboratory values.

**Conclusions:**

The VAS scores of the orthodontic (nonsurgical) group were significantly higher than those of the surgical group. No significant differences in VAS scores were found between the four surgical subgroups.

## 1. Introduction

Deformities in the maxillary and mandibular bones, including variations in form, size, and position, can cause abnormalities in the jaw relationship, resulting in malocclusion and other problems associated with facial deformities. Prognathism refers to protrusion of the maxilla or mandible or to protrusion of both jawbones concomitantly [1]. The two variations most commonly seen in Asian populations are mandibular prognathism and bimaxillary prognathism [2, 3]. Mandibular prognathism is characterized by the notable protrusion of one-third of the lower face, whereas bimaxillary prognathism signifies distinct protrusion of one-third of the middle face. In both cases, there is disharmony in the overall facial morphology, and this can further affect a patient psychologically.

Orthognathic surgery (OgS) is widely used to improve the appearance of facial protrusion and malocclusion. Although surgical precision is important, clinicians should also consider other factors (e.g., operation time, perioperative blood loss, and postoperative pain) to minimize the occurrence of subsequent complications and other sequelae [4–6]. Most patients are particularly concerned about postoperative pain. Therefore, to optimize the overall satisfaction of patients undergoing orthognathic surgery, it is critical to ensure that they fully understand and are mentally prepared for the surgery. Furthermore, patients with an abnormal facial profile must receive combination therapy with orthodontic treatment and orthognathic surgery. These two interventions can trigger varying levels of perceived pain among patients.

Patients who received orthodontic treatment without surgery were selected to form the control group (nonsurgical group), whereas other patients who underwent orthognathic surgery were selected as the surgical group. The variables assessed were sex, age, operation time, perioperative blood loss, and the postoperative change in blood components. The null hypothesis of this study proposed that the visual analog scale (VAS) scores of the first day posttreatment would be the same in the control group as in the surgical group. This study further compared the differences in perceived postoperative pain and other relevant variables for treating mandibular and bimaxillary prognathism by 4 surgical subgroups.

## 2. Materials and Methods

The sample is comprised of 100 patients who underwent only orthodontic treatment as the controls (i.e., nonsurgical group) and 100 patients who underwent orthognathic surgery (surgical group). Patients in the nonsurgical group received a fixed orthodontic appliance without being given any analgesics for pain management, and their VAS scores were recorded on the first day postintervention. The surgical group is comprised of 50 patients with mandibular prognathism and 50 patients with bimaxillary prognathism. The surgical procedures included intraoral vertical ramus osteotomy (IVRO), anterior segmental osteotomy of the mandible (ASO Md) and maxilla (ASO Mx), and genioplasty (GeP). The surgical group was further divided into four groups of 25 patients. The patients with mandibular prognathism formed Group 1 (IVRO alone) and Group 2 (IVRO+GeP). Those with bimaxillary prognathism formed Group 3 (ASO Mx+ASO Md) and Group 4 (ASO Mx+ASO Md+GeP). Groups 1 and 2 received six weeks of intermaxillary fixation treatment, whereas Groups 3 and 4 did not.

All patients underwent orthognathic surgery under hypotensive anesthesia. Data on the operation time, perioperative blood loss, and postoperative changes in blood components were examined. Postoperation pain management is based on a standardized protocol regarding the schedule of medicine administration. During hospitalization, an intravenous nonsteroidal anti-inflammatory drug (NSAID, Aspegic, 0.5 g) was prescribed for pain control at 6-hour intervals. The dose of NSAID is equally applied to the gender and surgical subgroups. On the first day posttreatment, the VAS scores (0–10 cm) of the nonsurgical group and the surgical group were recorded. SPSS Statistics, version 20 (SPSS Inc., Chicago, IL), was used for statistical analysis, and a *P* value < 0.05 was considered significant. Scores in the control and surgical groups were compared using *t*-tests; the null hypothesis was that there would be no differences in VAS scores on the first day posttreatment between these two groups. In addition, the VAS scores of 4 surgical subgroups were analyzed using *t*-tests, and Tukey's HSD (honest significant difference) test was used for post hoc analysis. Pearson's correlation coefficients were used to evaluate the correlations for the variables of sex, age, operation time, blood loss volume, and changes in blood components. The purpose of the present study is to compare the pain severity between the nonsurgical orthodontic group without pain medication and the orthodontic-OgS group with pain medication on day one after the initial treatment either via orthodontic force application or surgical intervention.

## 3. Results

Table 1 shows that our results revealed no significant differences between the nonsurgical and surgical groups for either sex or age. The female : male ratios of the two groups were 75 : 25 and 78 : 22, respectively, and the average age of the two groups was 26.1 and 25.4 years, respectively. Moreover, no significant differences were observed between the groups for the correlations of age and sex with the perceived postoperative pain levels. Where the first-day VAS scores for posttreatment pain were concerned, the nonsurgical group perceived a significantly higher level of pain than did the surgical group (mean VAS scores: 3.59 vs. 3.06 cm). The null hypothesis was rejected.

The values in Table 1 demonstrate that women in the nonsurgical group perceived a significantly higher level of pain on the first day posttreatment than those in the surgical group (mean VAS scores: 3.95 vs. 2.97 cm). However, men in the nonsurgical group perceived a significantly lower level of pain on the first day posttreatment than those in the surgical group (mean VAS score: 2.52 vs. 3.36). These results implied that the women who received orthodontic treatment perceived more intense pain than those who received orthognathic surgery, whereas their male counterparts perceived significantly greater pain in the surgical group than the nonsurgical group.

Within the surgical group, no significant differences were observed between the sexes in terms of age, operation time, blood loss volume, or reduction in postoperative hemoglobin (Hgb) and hematocrit (Hct) levels, as shown in Tables 2 and 3. In Tukey's honest significant difference test (Table 4), patients in Group 3 (mean age, 30 years) were significantly older than those in Group 4 (25.1 years), Group 1 (23.9 years), and Group 2 (22.9 years); the operation time for Group 1 (244.8 min) was significantly shorter than that for Group 2 (314.2 min), Group 3 (343.2 min), and Group 4 (391.2 min); the blood loss of Group 1 (107.8 mL) was significantly less than that of Group 3 (262.2 mL) and Group 4 (402.4 mL); and the postoperative decreases in Hgb and Hct levels in Group 1 (1.9 g/dL, 5.9%) were significantly lower than those in Group 3 (2.9 g/dL, 8.1%) and Group 4 (3.1 g/dL, 9.5%). When an intergroup (4 surgical subgroups) comparison was carried out, no significant differences were observed in the first-day postoperative VAS scores. The results laid out in Table 5 show that Pearson's correlation analysis of the four surgical subgroups revealed no association between each group's first-day postoperative VAS scores and sex, age, operation time, blood loss, or reduction in postoperative Hgb and Hct levels.

## 4. Discussion

Over the past decade, there has been an increasing trend emerging in the number of patients opting for surgical treatment to improve their malocclusion, a trend which may have been due to improved surgical techniques which alleviate perioperative and postoperative discomfort arising from the surgery. However, patients with maxillofacial abnormalities are often required to accept combination therapy with both orthodontic and orthognathic surgeries, which may involve different degrees of pain. Pain represents a highly complex response to intense stimuli and is accompanied by various effects on functioning. Importantly, pain is a highly subjective perception that differs significantly from person to person, and pain management has thus been a topic of considerable interest across the field of medicine.

During orthodontic treatment, pain is commonly observed as a side effect that varies according to the sex and age of patients, the magnitude and method of orthodontic force, and individual emotional responses to pain and tolerance of stress [7–10]. In the report of Kvam et al. [7, 9], 95% of patients presented pain during orthodontic treatment. After bonding fixed appliances, Kvam et al. [10] found that initial pain is perceived at posttreatment 2 h and peaks at 24 h. Fujiyama et al. [11] reported that the VAS score was more than 4 cm in treatment with the fixed appliance. In the present study, VAS of our patients was 3.59 cm. Postoperative pain can be caused not only by surgical wounds but also by neural injuries to the lingual nerve or inferior or superior alveolar nerves, surgical site inflammation, constrictive pain associated with soft tissue injury, and other problems induced by postoperative muscle and bone adaptation. This study focused mainly on patients who received nonsurgical orthodontic treatment and those who received orthognathic surgery and compared their perceived postoperative pain. Most patients who are offered orthognathic surgery usually accept orthodontic treatment, so a comparison of the results between the nonsurgical and surgical groups may be useful to inform future patients, helping to manage their expectations regarding the difference in the level of postinterventional pain (as well as other associated factors) which they might experience between orthodontic treatment and orthognathic surgery.

Because the two groups in this study showed no significant differences in sex or age, the results may be applicable to the general population. Notably, the first-day postintervention VAS scores of the nonsurgical group indicated that this group perceived a significantly higher level of pain than those who underwent orthognathic surgery. The possible reason for this outcome might be that no analgesic was given to the nonsurgical group, whereas the patients in the surgical group were periodically prescribed medication for pain relief. Another possible reason might be that the patients in the nonsurgical group may not have anticipated the degree of pain associated with fixed orthodontic appliances, which exert continuous force on the periodontal ligament through orthodontic tooth movement. This might suggest that the patients in the surgical group, who had undergone orthodontic treatment, were more mentally prepared for postoperative pain because they anticipated it prior to their surgery.

When stratified by sex, further analysis of the two groups revealed no difference in female : male ratios or age; however, differences in the VAS scores were noted between the two sexes in the nonsurgical group, where women perceived a significantly higher level of pain than did the men. Conversely, in the surgical group, no differences were observed in perceived pain levels between the sexes. Furthermore, when men and women in the nonsurgical group were compared with men and women in the surgical group, the analysis of the first-day posttreatment VAS scores revealed that women in the nonsurgical group reported a significantly higher level of pain than women in the surgical group, whereas the men in the surgical group reported a significantly higher level of pain than the men in the nonsurgical group.

Numerous studies [12, 13] have indicated the influence of postoperative satisfaction on patient stress levels. There have been assumptions that the greater preoperative stress levels a patient experiences, the greater his or her postoperative pain level will be. However, our findings reveal that women exhibited lower tolerance to the pain when the continuous orthodontic force was applied during initiation of the treatment, suggesting that they might be able to adapt mentally in their subsequent surgeries by accepting that postoperative pain would be an inevitable consequence. These results could be explored further to determine whether the preoperative stress levels between sexes differed significantly.

Niederhagen et al. [14] suggest that orthognathic surgery is the most postoperative pain among all oral and craniofacial surgeries, indicating that the duration of surgery and postoperative pain are closely correlated. In our study, operation-related factors (operation time, blood loss, and blood component reduction) and first-day postsurgical VAS scores were higher for the men than for the women. However, both male and female showed insignificantly in terms of age, operation-related factors, and first-day postsurgical VAS scores. Additionally, Tukey's honest significant difference test revealed that patients with bimaxillary protrusion (Groups 3 and 4) were significantly older than those with mandibular protrusion (Groups 1 and 2). This finding might imply that Asian patients have higher sensitivity toward visual abnormalities caused by mandibular protrusion, reverse overjet, and masticatory malfunction, whereas bimaxillary protrusion often has normal occlusion and does not affect masticatory function; hence, people with this condition may tend to postpone surgery until they are older.

Where the operation time and relevant blood components among the four groups were concerned, Group 4 demonstrated the largest and most significant changes. Comparisons of the operation time and blood loss during GeP surgery revealed that GeP surgery for mandibular prognathism had an operation time which was approximately 70 min longer and a 80 cc increase in blood loss; similarly, GeP surgery for bimaxillary protrusion showed an increase of approximately 50 min in operation time and a 140 cc increase in blood loss. The results of our study also demonstrated that there was greater blood loss during ASO for bimaxillary protrusion than that observed in bilateral IVRO. This might be because more bone marrow is involved in surgery to the maxillary and mandibular bones during ASO. No significant difference was observed in first-day postoperative pain between the four surgical groups.

The results of Pearson's correlation analysis of postoperative pain in each group challenged the generally held belief that first-day postoperative VAS scores correlate positively with operation time and blood loss; indeed, the results of the present study revealed no significant correlation in these variables among the four subgroups. This might be related to the sites of surgery. We presume that the prevalence of postoperative lower lip numbness in Groups 2, 3, and 4 may have been higher than that in Group 1 (from GeP surgery in Groups 2 and 4 and from anterior mandibular subapical osteotomy in Group 3). Lower lip numbness may relieve perceived pain even when considerable blood loss occurs, which may reduce the likeliness of observing a significantly positive relationship with the level of pain; by contrast, although the lowest amount of blood loss and lowest level of postoperative numbness were observed in Group 1, blood loss correlated significantly and positively with the level of pain.

Because tissue-level injury, inflammation, facial edema, and other harmful perioperative stimuli can affect the central nervous system, a variety of different methods and techniques can be used to control postoperative pain. Various research [15, 16] reports have indicated that patient-controlled analgesia (PCA) can control pain effectively following surgery; PCA allows patients to control the dose, and this helps to relieve postoperative anxiety and stress, which are the key factors contributing to postoperative pain. Similarly, related studies have shown that patients who have access to the PCA report improved levels of comfort and require shorter hospitalizations after orthognathic surgery. After assessing 45 patients who underwent orthognathic surgery, Evans et al. [17] found that their postoperative pain was not severe enough to require a high dose of narcotic analgesics. In our study, PCA was not used, but nonsteroidal anti-inflammatory drugs were prescribed to the patients who underwent surgery for pain control; the analgesic effect was similarly satisfactory.

The VAS is a unidimensional tool to measure pain intensity. However, pain is a multidimensional nature, made up of unpleasant sensory, emotional experience, cognitive, and behavioral elements. Therefore, VAS cannot reflect the overall aspects of a patient's pain experience. Without comparing the VAS baseline of a patient's pain tolerance, the present study revealed a weak point to interpret the difference of VAS scores between the control group and the surgical subgroups.

## 5. Conclusion

The results of this study demonstrate that the pain after orthognathic surgery, when appropriate analgesia is administered, is significantly lower than that from orthodontic treatment. Consequently, attaining a more complete understanding of orthognathic-orthodontic treatment with improved surgical techniques is expected to help meet patients' needs for preoperative psychosocial support and to reduce their postoperative pain.

## Figures and Tables

**Table 1:** Summary of all patient characteristics.

Variables	Control group	Surgical group	*P* value
Total patients	*n* = 100	*n* = 100	
Female (*n*)/male (*n*)	(75/25)	(78/22)	0.780
Age (y)	26.11 ± 6.17	25.44 ± 6.68	0.473
VAS (cm)	3.59 ± 1.79	3.06 ± 1.03	0.007^∗^
*P* value by gender	<0.001^∗^	0.191	
Female patients			
Age (y)	26.00 ± 6.35	25.81 ± 7.00	0.859
VAS (cm)	3.95 ± 1.84	2.97 ± 0.94	<0.001^∗^
Male patients			
Age (y)	26.4 ± 5.34	24.27 ± 5.21	0.178
VAS (cm)	2.52 ± 1.02	3.36 ± 1.29	0.019^∗^

*n*: number of patient; VAS: visual analog scale. ^∗^Chi-squared tests or two-sample *t*-test; statistically significant, *P* < 0.05.

**Table 2:** Summary of surgical patient characteristics by gender.

Variables	Female (*n* = 78)		Male (*n* = 22)		
	Mean	SD	Mean	SD	*P* value
Age (y)	25.81	7.00	24.27	5.21	0.267
Operation time (min)	319.68	80.20	336.36	104.42	0.493
Blood loss (mL)	218.14	185.02	316.36	344.41	0.210
Postoperative reduction					
Hgb (g/dL)	2.48	1.03	2.60	0.98	0.641
Hct (%)	7.54	3.16	7.92	2.79	0.592
VAS (cm)	2.97	0.94	3.36	1.29	0.191

*n*: number of patient; VAS: visual analog scale. Two-sample *t*-test; statistically significant, *P* < 0.05.

**Table 3:** Patient characteristics according to the four surgical subgroups.

Variables	Group 1		Group 2		Group 3		Group 4	
Gender (female/male)	*n* = 25		*n* = 25		*n* = 25		*n* = 25	
	(17/8)		(17/8)		(22/3)		(22/3)	
	Mean	SD	Mean	SD	Mean	SD	Mean	SD
Age (y)	23.9	4.42	22.9	3.78	30.0	8.74	25.1	5.93
Operation time (min)	244.8	43.94	314.2	69.98	343.2	75.46	391.2	73.38
Blood loss (mL)	107.8	58.41	186.6	135.66	262.2	218.06	402.4	308.44
Postoperative reduction								
Hgb (g/dL)	1.9	0.75	2.3	0.96	2.7	1.03	3.1	0.90
Hct (%)	5.9	2.11	7.0	2.87	8.1	3.08	9.5	2.87
VAS (cm)	3.1	1.14	3.0	1.22	2.9	0.71	3.2	0.95

*n*: number of patient; VAS: visual analog scale. Group 1: IVRO; Group 2: IVRO+GeP; Group 3: ASO Mx+ASO Md; Group 4: ASO Mx+ASO Md+GeP. Mx: maxilla; Md: mandible; IVRO: intraoral vertical ramus osteotomy; ASO: anterior subapical osteotomy; GeP: genioplasty.

**Table 4:** Four surgical groups in the Tukey HSD post comparison.

Variables	*F*	*P* value	Tukey HSD post comparison
Gender	1.98	0.122	—
Age (y)	6.582	<0.001^∗^	Group 3>4, 3>1, 3>2
Operation time (min)	20.107	<0.001^∗^	Group 4>2, 4>1, 3>1, 2>1
Blood loss (mL)	9.181	<0.001^∗^	Group 4>2, 4>1, 3>1
Postoperative reduction			
Hgb (g/dL)	7.202	<0.001^∗^	Group 4>2, 4>1, 3>1
Hct (%)	7.443	<0.001^∗^	Group 4>2, 4>1, 3>1
VAS (cm)	0.387	0.762	—

VAS: visual analog scale. ^∗^Statistically significant, *P* < 0.05; ^—^not significant. Group 1: IVRO; Group 2: IVRO+GeP; Group 3: ASO Mx+ASO Md; Group 4: ASO Mx+ASO Md+GeP. Mx: maxilla; Md: mandible; IVRO: intraoral vertical ramus osteotomy; ASO: anterior subapical osteotomy; GeP: genioplasty.

**Table 5:** Intragroup comparisons by Pearson correlation coefficient (*r*).

VAS (cm)	Group 1		Group 2		Group 3		Group 4	
	*r*	*P* value	*r*	*P* value	*r*	*P* value	*r*	*P* value
Gender	0.378	0.062	0.118	0.573	-0.284	0.169	0.190	0.364
Age (y)	0.217	0.298	-0.121	0.565	-0.361	0.077	0.007	0.974
Operation time (min)	0.016	0.938	0.029	0.892	-0.313	0.127	-0.354	0.082
Blood loss (mL)	0.382	0.600	-0.143	0.494	-0.359	0.078	-0.148	0.480
Postoperative reduction								
Hgb (g/dL)	0.215	0.303	-0.374	0.065	-0.236	0.256	0.069	0.744
Hct (%)	0.249	0.229	-0.321	0.117	-0.326	0.111	0.077	0.715

VAS: visual analog scale. ^∗^Statistically significant, *P* < 0.05; ^—^not significant. Group 1: IVRO; Group 2: IVRO+GeP; Group 3: ASO Mx+ASO Md; Group 4: ASO Mx+ASO Md+GeP. Mx: maxilla; Md: mandible; IVRO: intraoral vertical ramus osteotomy; ASO: anterior subapical osteotomy; GeP: genioplasty.

## Data Availability

The data used to support the findings of this study are available from the corresponding author upon request.
